# Current Status of Epidemiology, Diagnosis, Therapeutics, and Vaccines for the Re-Emerging Human Monkeypox Virus

**DOI:** 10.4014/jmb.2306.06033

**Published:** 2023-07-25

**Authors:** Wooseong Lee, Yu-Jin Kim, Su Jin Lee, Dae-Gyun Ahn, Seong-Jun Kim

**Affiliations:** Department of Convergent Research of Emerging Virus Infection, Korea Research Institute of Chemical Technology, Daejeon 34114, Republic of Korea

**Keywords:** Human monkeypox virus, epidemiology, diagnosis, therapeutics, vaccine

## Abstract

Monkeypox (Mpox) virus, a member of the *Poxviridae* family, causes a severe illness similar to smallpox, which is characterized by symptoms such as high fever, rash, and pustules. Human-to-human transmission cases have been reported but remained low since the first recorded case of human infection occurred in the Congo in 1970. Recently, Mpox has re-emerged, leading to an alarming surge in infections worldwide since 2022, originating in the United Kingdom. Consequently, the World Health Organization (WHO) officially declared the '2022–23 Mpox outbreak'. Currently, no specific therapy or vaccine is available for Mpox. Therefore, patients infected with Mpox are treated using conventional therapies developed for smallpox. However, the vaccines developed for smallpox have demonstrated only partial efficacy against Mpox, allowing viral transmission among humans. In this review, we discuss the current epidemiology of the ongoing Mpox outbreak and provide an update on the progress made in diagnosis, treatment, and development of vaccines for Mpox.

## Introduction

Monkeypox (Mpox), a virus-borne infectious disease that recently re-emerged worldwide, has raised concerns. It exhibits symptoms similar to those of smallpox, such as high fever, generalized body aches, and the development of distinctive rashes and pustules on the skin that last a few weeks [1]. It is caused by infection with Mpox virus (MPXV), which belongs to the *Orthopoxvirus* genus of the *Poxviridae* family, similar to the variola virus. MPXV was initially discovered in 1958 in laboratory monkeys during vaccine research, and was also discovered in a few rodent and small mammalian species. The first case of human infection was reported in the Congo in 1970, and human-to-human transmission cases have been reported since 1996, making Mpox a significant public health concern. Since 2022, an unusual increase in Mpox infection cases has occurred in Europe and North America, which are regions not associated with previously reported areas of Africa. In response, the WHO declared the '2022-23 Mpox outbreak' and initiated monitoring efforts [2]. Currently, Mpox is known to be a zoonotic disease, which means it can be transmitted through contact with the bodily fluids of MPXV-infected humans or animals in the vicinity or through bites or wounds from infected animals [3, 4].

MPXV is a large virus (200–250 nm in diameter) with a lipid envelope and a linear double-stranded DNA genome of approximately 197 kbp. The viral genome contains about 6.4 kbp inverted terminal repeats (ITR) and covalently closed hairpin ends. Genetic diversity at both ends affects the virulence, pathogenicity, and fatality of *Orthopoxviruses*. The central genomic regions covering open reading frames (ORFs) between C10L and A25R encode structural proteins and essential enzymes and are well conserved across other poxviruses.

The MPXV genome contains approximately 190 ORFs. However, the roles of many proteins, excluding certain key viral proteins involved in viral replication and assembly, are not yet clearly understood, although they are thought to be involved in host tropism and immunomodulation. The genome has a low GC content of approximately 33% and is characterized by the presence of numerous local tandem repeats, including the ITR. These features make it challenging to achieve a consistent reconstruction during gene replication and virion assembly [5].

**Fig. 1:**
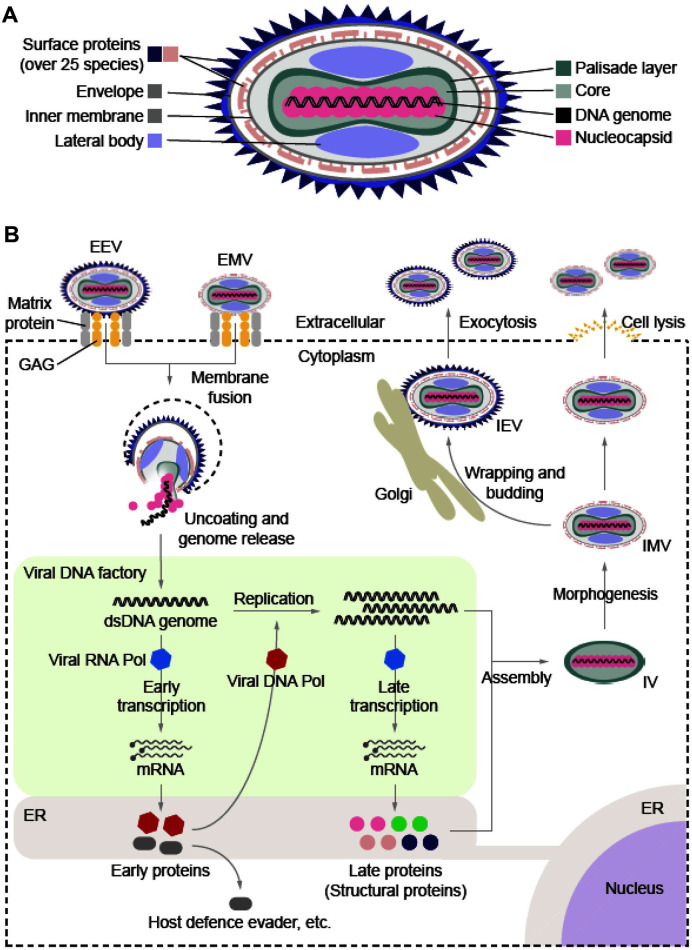
An overview of MPXV structure and lifecycle. (**A**) The virion mainly consists of DNA genome, nucleocapsids, dumbbell-shaped core, lateral bodies, inner membrane, envelope, and surface proteins. There are at least 25 species of surface proteins, which are distributed on envelope and/or inner membrane, respectively. (**B**) The attachment and membrane fusion of virions are dependent on the interaction among viral surface proteins, extracellular GAGs, and matrix proteins of the host cell. Uncoated genomic DNA immediately forms concentrated complex known as DNA factory to undergo the stages of transcription, replication, and translation, leading to the assembly of IV. Subsequently, morphogenesis occurs in the cytoplasm to shape IV into IMV. EVs with envelope formed in the Golgi complex are released from the cells through exocytosis, and IMVs without envelope can also be released from the cells through cell lysis. GAG, glycosaminoglycan; IV, immature virion; IMV, intracellular mature virion; IEV, intracellular enveloped virion; EEV, extracellular enveloped virion.

MPXV is composed of several constituents (genomic DNA, nucleocapsid, core, lateral body, inner membrane, envelope, and surface proteins) (Fig. 1A). The core is dumbbell-shaped and is surrounded by the core membrane, palisade layer, and lateral bodies.

MPXV entry occurs in a manner similar to the distinctive entry mechanism of other poxviruses; however, the detailed mechanism has not yet been fully elucidated. Herein, we briefly describe the basic entry process of vaccinia virus (VACV), which is a well-known poxvirus relatively, and the viral surface proteins involved, as well as the host receptors. It has been known that poxvirus has over 8 surface proteins on the envelope and over 20 surface proteins on the inner membrane. Adhesion to cellular receptors, membrane fusion, and inter-host transmission occur through parallel interactions between these surface proteins and their respective host receptors. Fortunately, surface proteins among poxviruses exhibit approximately 93-98% sequence similarity based on comparison between vaccinia virus (VACV) and MPXV [6], suggesting similar roles in the entry process. Firstly, certain surface proteins located on the envelope bind to the host's extracellular glycosaminoglycans (GAGs), inducing cell adhesion [7-9]. D8L (E8L of MPXV) binds to chondroitin sulfate, while A27L (A29L of MPXV) and H3L (H3L of MPXV) bind to heparan sulfate. Additionally, A26 interacts with laminin. The receptor bound by L1R (M1R of MPXV) remains unidentified, and the viral protein that is expected to bind to the macrophage receptor with collagenous structure (MARCO) has not been identified yet. After each adhesion, A34 and B5 (B6 of MPXV) induce the rupture of the envelope membrane, exposing the inner membrane [10]. On the other hand, it has been revealed that not all of the proteins involved in cell adhesion are indispensable for infection [9]. The exposed inner membrane forms hemifusion state under lower pH conditions with a host membrane, and that caused by entry fusion complex (EFC) constructed by various viral surface proteins such as A16, A21, A28, F9, G3, G9, H2, J5, L1, and L5 [9, 11]. Subsequently, the entry of the MPXV core is completed through the membrane pore formed in the hemifusion portion (Fig. 1B). The released MPXV genomic DNA forms concentrated complex known as DNA factory immediately to undergo the stages of transcription, replication, and translation, leading to the assembly of immature virions (IV), which then become intracellular mature virions (IMV) through morphogenesis in cytoplasm (Fig. 1B) [12, 13]. Depending on the formation of a double-membrane envelope in the Golgi, the virus can escape in two forms: the intracellular enveloped virion (IEV), which is released outside the cell through exocytosis, and IMV, which can also be released without enveloping through host cell lysis (Fig. 1B). Although the surface glycoprotein, envelope composition, and receptor interaction mechanisms are not fully understood, both viral forms are infectious to neighboring host cells [11, 14].

In this review, we compiled information on the global epidemiology, diagnosis, treatment, and vaccine development status following the Mpox outbreak. In order to countermeasure Mpox outbreak, we have also summarized the content related to specific therapeutic agents and vaccine candidates for Mpox. This review will contribute to the early stages of basic research and antiviral studies related to MPXV and provides assistance in preparing for pandemic outbreaks caused by novel MPXV variants.

## Epidemiology

**Fig. 2:**
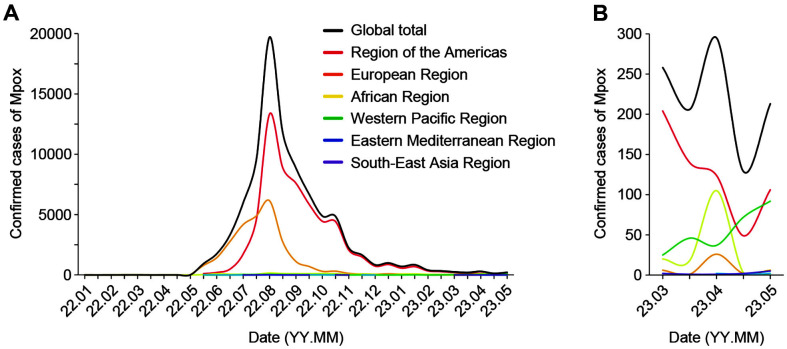
Epidemic curves of the confirmed Mpox cases. The presentation of the case counts is divided into overall data from Jan 1, 2022, to May 10, 2023 (**A**) and recent data from Mar 1, 2023, to May 10, 2023 (**B**). All the results were calculated and visualized based on half the value of the month.

As of May 16, 2023, 87,479 confirmed cases of Mpox and 1,095 probable cases, including 140 deaths, were reported globally by the WHO [2]. The global epidemic curve peaked in August 2022, with weekly reported cases over 7,000 (Fig. 2A). Since then, there has been a gradual decrease, and as of May 2023, approximately 100 weekly cases have been recorded. Among the confirmed cases, the United States reported the highest number (59,343), followed by Europe (25,888). The African region had a relatively low number of cases (1,592). Notably, the western Pacific region showed a gradual increase in cases since March 2023 (Fig. 2B), with approximately 40 weekly cases recorded. Australia (145), Japan (135), China (87), and South Korea (75) accounted for the majority of cases, with China and Korea experiencing an increase of cases since April 2023.

Of all the WHO-reported types of transmission, sexual encounters were the most common (82.0%). Of all confirmed cases with available data, 96.2% were male and the median age was 34 years. Among those with known data on sexual orientation, 84.1% were categorized as men who had sex with men. In addition, 52.3% of the cases with available HIV status were HIV-positive.

Thus far, two major genotypes of MPXV have been identified. The genotype originating in Central Africa (the Congo Basin) has been classified as Clade I, while the one originating in West Africa has been designated as Clade II, which was subsequently renamed as Clade IIa after the global outbreak. Clade I has been reported to have a higher infectivity and fatality rate than Clade II (10.6%:3.6%) [15]. This was attributed to genomic deletions and fragmentations in the ORF of Clade II, which led to a decrease in virulence. In the case of Clade I, it is based on the inhibition of T-cell activation, which suppresses the expression of pro-inflammatory cytokines [1, 16]. The current world outbreak (2022–2023) was identified as Clade IIb, a subtype of the West African clade with low severity, presumably exported out of Africa and amplified by travelers who had sexual contact with men [2]. Currently, most infected people belong to the B.1 lineage of Clade IIb, and some belong to the A.2 lineage. Lineage B.1 has accumulated over 50 nucleotide changes compared to a Clade IIb MPXV isolated in 2018 [17]. Although the total fatality rate of cases counted after the outbreak was significantly lower at 0.16% (deaths/confirmed cases:140/87,479), these many nucleotide changes are likely to have influenced the activity alteration of APOBEC3 cytosine deaminase and the substantial increase in human-to-human transmission [18].

## Diagnosis

The analytical methods developed for the detection of MPXV are typically conducted in biosafety level 3 facilities and are similar to the methods for other viruses. The most efficient method for sample preservation and fast analysis involves real-time polymerase chain reaction (PCR) using viral DNA isolated from lesion tissue. Since MPXV is rarely detected except during the short period of initial infection, the PCR analysis using serum samples is not recommended by the WHO [19]. Additionally, methods for detecting viral proteins and antibodies in serum are prioritized and widely utilized. Electron microscopy analysis of viruses isolated from clinical specimens and immunohistochemistry of tissue samples are also available, but they have limitations, as they require skilled techniques and specialized analytical equipment.

The diagnostic targets of MPXV are diverse. Among MPXV genes, the D6R gene of MPXV is a conserved region (> 100 nucleotides in length) that spans the entire *Poxviridae* family. Therefore, it has been utilized as a target for pan-poxvirus detection by real-time PCR [20]. The E7R gene, which encodes the DNA-dependent RNA polymerase subunit 18 (rpo18), and the E9L gene, which encodes the viral DNA polymerase, are known to have conserved primer targets within the *Orthopoxvirus* genus [21, 22]. The B6R gene, which encodes an extracellular envelope protein, is a specific target of MPXV [22]. B7R [23], F3L, and N3R genes [24] are also genes of interest for diagnosis based on PCR, as they are highly conserved among MPXV. The current WHO guideline considers any PCR set with an appropriate control group to be acceptable regardless of the primer target [19], and the U.S. CDC employs a primer set targeting G2R as the standard for detection [25, 26].

As an immunological method in clinical area, enzyme-linked immunosorbent assay (ELISA) which has been developed to detect patient antibodies (IgG, IgM) against Orthopoxvirus is still used to MPXV as well in the serum at five days or more than eight days after the onset of rash [27, 28]. However, the detection specificity of these antibodies is insufficient to distinguish because of the highly conserved antigen epitopes between MPXV and other poxviruses [1]. In addition, ELISA for detecting IgG and IgM has the disadvantage of being unable to distinguish between people infected with other poxviruses in the past, people who have been vaccinated, and people infected with Mpox [1, 27, 29]. Consequently, the use of this method is limited to epidemiologic studies [30].

## Antiviral Therapies

Currently, no specific antiviral therapy has been approved for treating human MPXV infections. During the ongoing Mpox outbreak, supportive care and pain control are recommended as the initial treatment options for most patients. Owing to their broad-spectrum antiviral activity, several therapies (cidofovir, brincidofovir, tecovirimat, and vaccinia immunoglobulin) for smallpox infection are considered for the treatment of severe cases of Mpox and high-risk patients such as immunocompromised people, infants, pregnant or breastfeeding women, and people with skin integrity issues (Treatment Information for Healthcare Professionals from CDC [31]).

A viral replication step has been the most important antiviral target as nucleotide analogs can interfere synthesis of viral genomes. Cidofovir, an FDA-approved drug used to treat cytomegalovirus infections, inhibits DNA synthesis and interferes with replication of various DNA viruses. Owing to its broad-spectrum activity, cidofovir is considered a potential therapeutic option against other DNA viruses, including orthopoxviruses. Cidofovir remains an investigational treatment option for smallpox [32] and Mpox. Brincidofovir is a cidofovir prodrug that contains a lipid conjugate for better membrane permeability in infected cells [33]. Brincidofovir showed higher protective efficacy than cidofovir against various DNA viruses, such as orthopoxviruses [34], herpesviruses [35], and adenoviruses [36]. Recently, FDA approved brincidofovir for the treatment of smallpox. Both cidofovir and brincidfovir are nucleoside analogs that are incorporated into newly synthesized DNA strands, terminating DNA chain elongation [37].

Viral envelope is another important antiviral drug target as it plays a critical role not only in maintaining virion and protecting from diverse environment but also in viral entry. Tecovirimat, another FDA-approved drug used to treat smallpox, targets a specific orthopoxvirus membrane protein p37 (F13L of VACV), which is responsible for forming the envelope of immature intracellular virus particles [38], eventually interfering with cell-to-cell and systemic virus transmission [37, 39]. Since p37 is highly conserved in orthopoxviruses, the p37-targeting drug tecovirimat is considered a potential therapeutic option for ongoing Mpox outbreaks and is currently undergoing various clinical trials.

Each stage of the viral life cycle may be considered a potential target for the development of new antiviral drugs. Many investigational anti-orthopoxviral drugs that target key components at different stages, from viral entry to progeny release, have been identified (Table 1).

I. Entry and uncoating

• Vaccinia immune globulin [40, 41]: Neutralizing activity of antibodies blocks the interaction between virions and host receptors, inhibiting viral entry.

II. Early transcription

• Adenosine N1-oxide [42]: Incorporation of adenosine N1-oxide into early viral transcripts interferes with the translation of viral mRNA, not cellular mRNA, blocking early viral gene expression.

• Nigericin [43]: Although its precise mechanism of action remains unknown, nigericin inhibits viral replication at the early transcription stage. As a carboxylic ionophore, nigericin can be readily incorporated into biological membranes to stimulate the exchange of monovalent cations with protons. These environmental changes may affect the viral mRNA synthesis.

• Aurintricarboxylic acid [44]: Aurintricarboxylic acid inhibits the transcription of early viral genes by targeting both the cellular (blocking the extracellular signal-regulated kinase 1/2 (ERK1/2) signaling cascade, which is essential for VACV replication) and viral factors (inhibiting the phosphatase activity of the viral enzyme H1L, which is required to initiate viral transcription).

III. DNA synthesis

• Nucleoside analogs: Nucleoside analogs mimic natural nucleosides to compete with natural nucleosides during DNA synthesis and are incorporated into newly synthesized DNA strands. Once incorporated, nucleoside analogs terminate the DNA chain elongation. Numerous nucleoside analogs have been identified as antiviral therapeutics, and several compounds have been confirmed to induce broad-spectrum anti-orthopoxviral activities (listed below).

◆ Cidofovir & brincidofovir [37, 45]

◆ N-methanocarbathymidine [46]

◆ Aphidicolin [47]

◆ Cytosine arabinoside & phosphonoacetic acid [48]

◆ 1-(2-deoxy-4-thio-beta-d-ribofuranosyl)-5-iodouracil (4'-thioIDU) [49]

◆ 5-substituted deoxyuridine analogs [50]: Viral thymidine kinase phosphorylates these modified molecules, enhancing their antiviral activities.

• Small peptide aptamers [51, 52]: Peptide aptamers targeting A20, a major component of the viral replication complex, impair viral DNA synthesis.

• Hydroxyurea [53] is a ribonucleotide reductase inhibitor that limits deoxyribonucleotide production, thereby inhibiting viral DNA synthesis.

IV. Late transcription

• Isatin-beta-thiosemicarbazone (IBT) [54, 55]: The precise mechanism of action of IBT is not completely understood. However, IBT terminates late viral gene transcripts by degrading late viral mRNAs, followed by the cessation of late viral protein production. Moreover, IBT indirectly regulates viral transcription elongation factors [56].

• Methisazone (N-methyl-isatin-/i-thiosemicarbazone, N-MeIBT,MelBT) [57]: Methisazone inhibits mRNA and protein synthesis and has antiviral activity. Methisazone has been reported to be effective in smallpox prophylaxis.

• Ethacrynic and alpha-lipoic acid [58]: Both ethacrynic and alpha-lipoic acids inhibit vaccinia late gene expression, but not viral entry, early gene expression, and viral DNA synthesis.

V. Assembly

• Rifampicin [59, 60]: Rifampicin interferes with the formation of viral membranes during the early assembly step, resulting in the inhibition of viral morphogenesis.

• Mitoxantrone [61, 62]: As a DNA ligase inhibitor, mitoxantrone blocks virion assembly, but its protective efficacy is limited in vivo.

• Ofloxacin [63]: Ofloxacin inhibits topoisomerase, which is essential for virion assembly.

• Novobiocin [64, 65]: Novobiocin is also a topoisomerase inhibitor, interfering with virion assembly.

VI. Maturation

• TTP-6171 [66]: TTP-6171 inhibits I7L core proteinase, a cysteine proteinase that processes viral structural and membrane proteins, and blocks the maturation of intracellular virions.

• Recently, a computer modeling strategy identified several potential protease inhibitors with better binding affinity than that of TTP-6171 [67]: (1) Gallicynoic Acid F (NPA002071), (2) H2-Erythro-Neopterin (NPA000530), (3) Nigcollin C (NPA029767), (4) NPA24545, and (5) Vaccinol M (NPA030378)

VII. Secondary envelopment

• Tecovirimat [68]: A VP37-targeting agent tecovirimat inhibits the formation of enveloped viral particles. Formation of this envelope is important for viral egress and the next round of infection in other cells. Accordingly, targeting VP37 with a tecovirimat prevents the viral spread to other cells in the body. Its protective efficacy against the circulating Mpox strain has been confirmed in vitro [69] and clinical trials are in progress.

• N1-isonicotinoyl-N2-3-methyl-4-chloro-benzoylhydrazine (IMCBH) [70, 71]: IMCBH is another agent that affects secondary envelope formation by preventing the wrapping of intracellular virions by Golgi membranes.

VIII. Release of progeny virus particles

• Imatinib [72-74]: As a host-targeting agent, imatinib inhibits cellular Abl family tyrosine kinases, which are required for the release of infectious progeny viral particles.

• Terameprocol [75]: Terameprocol interferes the formation of actin tails which are crucial for spreading viruses to neighboring cells.

• Host targeting drug, nitroxoline [76]: Although its mechanism of action is not known, nitroxoline inhibits the phosphoinositide 3‐kinase/protein kinase B/mammalian target of rapamycin (PI3K/AKT/mTOR) signaling pathway and stimulates the fibrosarcoma/mitogen‐activated protein kinase/extracellular signal‐regulated kinase (RAF/MEK/ERK) signaling pathway. Both signaling cascades are involved in orthopoxvirus replication [77, 78].

## Vaccines

The currently available smallpox vaccine Dryvax, a live VACV that protects humans from both smallpox and Mpox, is a cloned vaccine strain [79]. However, large doses of these vaccines can cause serious side effects in high-risk individuals with acquired or congenital immune system defects [80]. Interestingly, the main mode of protection of an unattenuated smallpox vaccine against Mpox is mediated by neutralizing antibodies (Abs) [81]. Unattenuated Dryvax, modified vaccinia Ankara (MVA), and NYVAC do not protect against lethal Mpox in immunodeficient animals infected with the simian immunodeficiency virus (SIV) [82]. This was confirmed to be due to defective maturation of high-affinity protective Abs under CD4+ T cell depletion. LC16m8 [83], a VACV-mediated vaccine attenuated by passaging in mammalian primary cell, and VACΔ6 [84], a VACV-mediated vaccine intentionally modified six genes, also remain in phase 2 and 1 clinical trials respectively as a vaccine against MPXV yet (All clinical status was referenced in the ‘WHO Mpox Vaccine Tracker’ updated on April 24, 2023 [85]).

Orthopoxviruses, a family of double-stranded DNA viruses, comprise a variety of viruses, including human MPXV, variola virus, VACV, and cowpox virus [86]. Orthopoxvirus infection and immunization can confer immunity to other viruses, and as a result, two vaccines have been approved by the FDA to prevent MPXV [87]. ACAM2000 is a second-generation live VACV vaccine, and Bavarian Nordic (JYNNEOS, also known as MVA-BN, Imvamune, or Imvanex) was developed as a modified vaccinia Ankara (MVA)-based attenuated third-generation smallpox vaccine [88]. Since approval, the indication has been expanded based on the data from the MPXV challenge study conducted in non-humans (FDA's authorized “JYNNEOS” vaccine for counteracting Mpox global public health emergency). These two VACV-based smallpox vaccines cross-protect against MPXV [6]. Unfortunately, smallpox vaccines do not completely protect against circulating MPXV [89]. ACAM2000 is a highly regenerated VACV vaccine with serious side effects [90] (Table 2). In contrast, JYNNEOS, which is based on a live attenuated orthopoxvirus, is expected to have the lowest incidence of severe adverse events as a replication-deficient vaccine [90].

## Development of Novel Mpox Vaccines

Currently, mRNA- and DNA-based vaccines are being developed. Among them, one of the mRNA vaccine is the combination of mRNA-A-LNP and mRNA-B-LNP which are designed to contain two IMV-specific proteins (A29L and M1R) and two infected EEV-specific proteins (A35R and B6R) of MPXV, respectively [91]. Combinations of IMV- and EEV-specific immunogens have been studied to provide more protection than immunogens alone [92]. In a recent study, the immunogenicity of a multivalent mRNA vaccine candidate, MPXVac-097 (A29L, A35R, M1R, E8L, and B6R linked to a 2a peptide), was characterized in a mouse model [93]. MPXVac-097 elicits MPXV-specific T-cell responses and neutralizes VACV infections. As other effective mRNA vaccine candidates against MPXV, Rmix4 and Rmix6 encode each four (M1R, A29L, B6R, A35R) and six (M1R, H3L, A29L, E8L, B6R, and A35R) of MPXV antigens [94]. Freyn *et al*. also focused on same antigens (M1R, A29L, B6R, A35R) to develop a mixture vaccine of 4 mRNA-LNPs [95]. VGPox 1 and VGPox 2 express similar fusion proteins composed of extracellular domain of A35R and a full length M1R of MPXV [96].

Alternatively, several VACV genes and gene combinations have been tested for their immunogenicity and protective efficacy in mice using a gene gun delivery DNA vaccine. [97]. The four-gene combination DNA vaccine showed immunogenicity in non-human primates and protected 100% of the VACV-infected mice. DNA vaccines (expressing A27L, A33R, L1R, and B5R), named 4pox, have been reported to protect against fatal Mpox, rabbit pox, and VACV infections [98, 99]. Interestingly, vaccination of plasmid DNAs encoding these four antigens (L1R, A27L, A33R, and B5R) with boosting by the equivalent recombinant proteins showed much improved vaccine efficacy against MPXV [100]. In addition, Hirao *et al*. have reported pre-clinical data of a multivalent DNA vaccine comprising 8 VACV genes (A4L, A27L, A33R, A56R, B5R, F9L, H3L, and L1R) [101].

Franceschi *et al*. demonstrated the effective anti-MPXV properties of a vaccination involving three recombinant bovine herpesvirus 4 (BoHV-4) vectors. These vectors were designed to express the MPXV A29L, M1R, and B6R proteins, and the combination of them yielded positive results in animal tests [102].

Conventional recombinant protein-based vaccines are currently being developed. Because live poxviral vectors express many cytokines and chemokines, it is difficult to analyze the effects of immunomodulatory approaches [103]. On the other hands, vaccination of the recombinant subunit platform comprising A33R, B5R, L1R, and A27L of VACV with Alum adjuvant and CpG was reported successful IgG generation and full protection against VACV, even in a single-targeting vaccination trials [104, 105]. This study presents a simple subunit-based vaccine with excellent potential to increase immunogenicity and protective efficacy in high-risk populations.

Synthetic vaccines have recently been proposed as new tools for controlling infectious diseases. The novel immunogens containing a mixture of synthetic peptides, which were site-specifically designed for epitopes exposed on the multiple proteins of pathogen, has been shown to be an efficient approach as vaccine development [106]. Importantly, these synthetic vaccines promote the production of antibodies that neutralize the viral proteins when administered in monkeys and were able to delay or suppress the disease development in immunized human volunteers [107]. This strategy can be reliably used for diseases caused by viruses such as MPXV by including immunogenic epitopes and appropriate immune stimulants [108]. This allows development at a relatively low cost, particularly owing to low biosafety requirements, and presents remarkable immune diversity [109].

## Summary

Currently available therapeutics and vaccines for smallpox are cross-reactive to MPXV [6, 31, 32]. However, there are limitations to the therapeutic efficacy of different treatment and vaccine products against Mpox; thus, the development of specific therapeutics and vaccines against Mpox is still required [89]. One of them is that VACV exist in two forms (IMV and EEV) and infect cells by different mechanisms [11]. Thus, it is highly recommended to develop antiviral drugs targeting the replication steps of the viral life cycle, which can inhibit viral propagation regardless of viral entry. For example, tecovirimat does not affect the intracellular form of the virus and can only inhibit the formation of enveloped virions because it targets the p37 membrane protein of VACV, which only exists in EEV [68, 110]. In contrast, cidofovir and brincidofovir block orthopoxvirus DNA polymerase-mediated DNA synthesis, inhibiting viral replication [37, 45].

As for the vaccine development, multivalent vaccines created by the addition of MPXV immunogens to previously developed vaccine components are key strategies for the development of Mpox vaccines. Since two forms of VACV (i.e., IMV and EEV) can enter cells by different mechanisms, the composition of immunogens may affect their neutralizing activity. Indeed, despite the high immunogenicity, sera from animals immunized with the EEV proteins did not show neutralizing activity against IMV. More importantly, protective efficacy was better in animals immunized with combinations of IMV and EEV proteins than in animals immunized with individual IMV or EEV proteins [111]. Hence, this should be considered in the development of multivalent Mpox vaccines.

Another key strategy for improving vaccines is to strengthen previously developed vaccines to cover more diverse strains of orthopoxviruses, including Mpox. This is possible by boosting cellular immune responses. Since Mpox has highly similar amino acid sequences to vaccinia viruses, T cell-mediated cellular responses induced by VACV vaccines are cross-reactive to Mpox [112]. Optimization of adjuvants or the addition of peptides conserved between the VACV and Mpox can increase T cell-mediated cellular responses, allowing vaccines to target a broad range of viruses.

Together with the development of rapid diagnostics specific for Mpox, improvements in the effectiveness of therapeutics and vaccines against Mpox will prevent future Mpox outbreaks.

## Figures and Tables

**Fig. 3:**
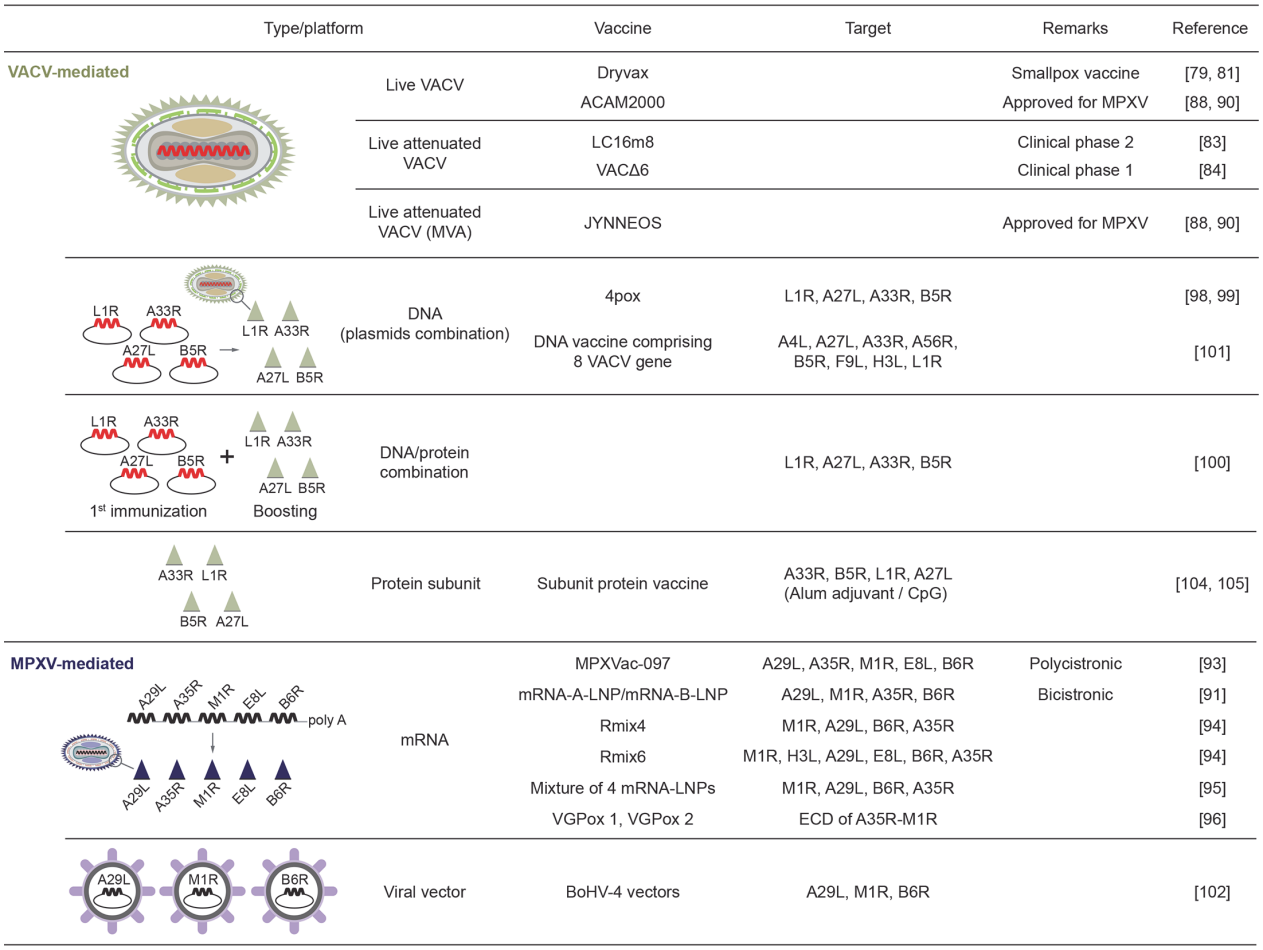
The characteristics of vaccine candidates against Mpox.

**Table 1:** Candidate anti-orthopoxvirus agents with defined targets.

Stage of life cycle	Candidate agent	Mechanism of action	Stage of development	Ref
Entry	Vaccinia immune globulin	Neutralizing viral particles	Approved for vaccinia infection	[40, 41]
Early transcription	Adenosine N1-oxide Nigericin Aurintricarboxylic acid	Inhibiting viral early gene transcription	Preclinical research	[42] [43] [44]
DNA synthesis	Cidofovir	Nucleoside analogs	Available through IND* protocol for smallpox	[37]
	Brincidofovir		Approved for smallpox / Clinical trial phase 3 for dsDNA viruses including MPOX	[45]
	N-Methanocarbathymidine Aphidicolin Cytosine arabinoside Phosphonoacetic acid 4'-thioIDU 5-substituted deoxyuridine analogs		Preclinical research	[46] [47] [48] [48] [49] [50]
	Small peptide aptamers	Binding to virus replication complex	Preclinical research	[51, 52]
	Hydroxyurea	Ribonucleotide reductase inhibitor	Preclinical research	[53]
Late transcription	IBT Methisazone Ethacrynic acid Alpha-lipoic acid	Inhibiting viral late gene transcription	Preclinical research	[54, 55] [57] [58] [58]
Assembly	Rifampicin	Interfering formation of viral membrane	Preclinical research	[59, 60]
	Mitoxantrone	DNA ligase inhibitor	Preclinical research	[61, 62]
	Ofloxacin Novobiocin	Topoisomerase inhibitor	Preclinical research	[63] [64, 65]
Maturation	TTP-6171	Cysteine proteinase inhibitor	Preclinical research	[66]
Secondary envelopment	Tecovirimat	VP37 inhibitor	Approved for smallpox / Clinical trial phase 3 for MPOX	[68]
	IMCBH	Preventing intracellular virion wrapping	Preclinical research	[70, 71]
Egress	Imatinib	Inhibiting cellular Abl-family tyrosine kinases	Preclinical research	[72-74]
	Terameprocol	Inhibiting actin tail formation	Preclinical research	[75]
	Nitroxoline	Inhibiting the signal cascade that modulates virus replication	Preclinical research	[76-78]

*Investigational new drug (IND)

**Table 2:** Approved vaccines to prevent MPXV infection.

Trade name	Manufacturer	Molecule type	Target disease	Inoculation site	Dosing interval and number	Injection volume	Side effects
JYNNEOS/ Imvanex	Bavarian Nordic AS	Live Attenuated Vaccine	Monkeypox; smallpox; vaccinia virus (EU only)	Subcutaneous Intradermal	2 doses administered 28 days (4 weeks) apart	0.5 ml 0.1 ml	Pain at the injection site, redness, itching, fever, headache, tiredness, nausea, chills, and muscle aches
ACAM2000	Gaithersburg, Inc.	Live virus Vaccine	Smallpox	Percutaneous	Single dose using a bifurcated needle	0.0025 ml droplet of reconstituted vaccine (100 doses)	Itching, sore arm, fever, headache, body ache, mild rash, fatigue, myocarditis, and pericarditis
